# Efficacy, dose–response, and aerosol delivery of dry powder synthetic lung surfactant treatment in surfactant-deficient rabbits and premature lambs

**DOI:** 10.1186/s12931-022-02007-8

**Published:** 2022-04-04

**Authors:** Frans J. Walther, Alan J. Waring, Monicah Otieno, Robert M. DiBlasi

**Affiliations:** 1 Department of Pediatrics, David Geffen School of Medicine, University of California Los Angeles, Los Angeles, CA 90095 USA; 2 Lundquist Institute for Biomedical Innovation at Harbor-UCLA Medical Center, 1124 W Carson Street, Torrance, CA 90502-2006 USA; 3 Department of Medicine, David Geffen School of Medicine, University of California Los Angeles, Los Angeles, CA 90095 USA; 4 Department of Translational Discovery, Nonclinical Development, Bill & Melinda Gates Medical Research Institute, Cambridge, MA 02139 USA; 5 Respiratory Therapy Department, Seattle Children’s Hospital, Seattle, WA 98105 USA; 6 Center for Integrative Brain Research, Seattle Children’s Research Institute, Seattle, WA 98101 USA

**Keywords:** Dry powder (DP) Synthetic lung surfactant, Surfactant protein B (SP-B), Super Mini-B (SMB) peptide, B-YL peptide, Lactose, Trehalose, Low flow aerosolization chamber (LFAC), Respiratory distress syndrome (RDS), Surfactant-deficient rabbits, Premature lambs, Noninvasive ventilation

## Abstract

**Background:**

Dry powder (DP) synthetic lung surfactant may be an effective means of noninvasive delivery of surfactant therapy to premature infants supported with nasal continuous positive airway pressure (nCPAP) in low-resource settings.

**Methods:**

Four experimental DP surfactant formulations consisting of 70% of phospholipids (DPPC:POPG 7:3), 3% Super Mini-B (SMB) or its sulfur-free derivate B-YL as SP-B peptide mimic, 25% of lactose or trehalose as excipient, and 2% of NaCl were formulated using spray drying. In vitro surface activity was confirmed with captive bubble surfactometry. Surfactant particle size was determined with a cascade impactor and inhaled dose was quantified using a spontaneously breathing premature lamb lung model supported with CPAP. In vivo surfactant efficacy was demonstrated in three studies. First, oxygenation and lung compliance were monitored after intratracheal instillation of resuspended DP surfactant in intubated, ventilated, lavaged, surfactant-deficient juvenile rabbits. In dose–response studies, ventilated, lavaged, surfactant-deficient rabbits received 30, 60, 120 or 240 mg/kg of DP B-YL:Lactose or B-YL:Trehalose surfactant by aerosol delivery with a low flow aerosol chamber via their endotracheal tube. Noninvasive aerosolization of DP B-YL:Trehalose surfactant via nasal prongs was tested in spontaneous breathing premature lambs supported with nCPAP. Intratracheal administration of 200 mg/kg of Curosurf^®^, a liquid porcine surfactant, was used as a positive control.

**Results:**

Mass median aerosol diameter was 3.6 μm with a geometric standard deviation of 1.8. All four experimental surfactants demonstrated high surface efficacy of intratracheal instillation of a bolus of ~ 100 mg/kg of surfactant with improvement of oxygenation and lung compliance. In the dose–response studies, rabbits received incremental doses of DP B-YL:Lactose or B-YL:Trehalose surfactant intratracheally and showed an optimal response in oxygenation and lung function at a dose of 120–240 mg/kg. Aerosol delivery via nasal prongs of 1 or 2 doses of ~ 100 mg/kg of B-YL:Trehalose surfactant to premature lambs supported with nCPAP resulted in stabilization of spontaneous breathing and oxygenation and lung volumes comparable to the positive control.

**Conclusion:**

These studies confirm the clinical potential of DP synthetic lung surfactant with B-YL peptide as a SP-B mimic to alleviate surfactant deficiency when delivered as a liquid bolus or as an aerosol.

## Background

Lung immaturity is associated with deficient production of lung surfactant and is the main reason for early neonatal respiratory failure in very premature infants [1]. Mammalian lung surfactant is produced by alveolar type 2 cells and consists of a mixture of phospholipids and four surfactant proteins. Dipalmitoylphosphatidylcholine (DPPC) and phosphatidylglycerol (PG) are essential phospholipids, surfactant proteins A and D are important for native immunity, and surfactant proteins B and C (SP-B and SP-C) are surface active. The presence of SP-B in the lung is pivotal as hereditary deficiency is lethal in animals and humans [2]. The introduction of animal-derived lung surfactant in the 1990s has dramatically improved survival of very premature infants [3], but the high costs of porcine and bovine lung surfactant production have led to a search for an advanced synthetic lung surfactant formulation based on SP-B and/or SP-C peptide analogs in a phospholipid mixture [4]. However, all these surfactant preparations require intratracheal administration, whereas respiratory support of premature infants has gradually shifted from invasive to noninvasive support, and especially to nasal continuous positive airway pressure (nCPAP) [5, 6] to minimize lung injury and chronic lung disease. Although noninvasive respiratory support can be assisted with surfactant instillation via a thin tracheal catheter, i.e., the less invasive surfactant administration (LISA) approach [7], aerosol delivery of dry powder (DP) synthetic lung surfactant better fits into a noninvasive respiratory support approach and eases surfactant administration to premature infants, especially those treated in a low-resource setting.

Recent work by our group explored noninvasive administration of a DP synthetic lung surfactant in two surfactant‐deficient animal models, i.e., lavaged juvenile rabbits and premature lambs [8]. The surfactant formulations tested consisted of two or three phospholipids, the SP-B peptide mimic Super Mini-B (SMB) [9] and the excipient polyglycitol SD-30 (Stabilite™, Grain Processing Corp., Muscatine, IA 52761) and showed proof-of-principle of this noninvasive approach. Surfactant was delivered with a Low Flow Aerosolization Chamber (LFAC: Acorda Therapeutics, Waltham, MA 02451, USA) and a 30 mL bellow bottle. Following these initial studies, significant formulation development work was conducted to optimize the aerosol properties, stability, and suitability of the DP synthetic lung surfactant composition [10].

Here, we compare the in vivo surface activity of a set of DP lung surfactant formulations that may be suitable for noninvasive clinical treatment in a low-resource setting. Based on a recent in vitro study [10], we chose a 7:3 ratio of DPPC and 1-palmitoyl 2-oleyl PG (POPG) as phospholipid mixture and SMB and B-YL as candidate SP‐B peptide mimics [9, 11]. SMB is a 41‐residue peptide based on the N‐ and C‐terminal regions of SP‐B, covalently joined with two disulfides bridging a hairpin turn [9]. B‐YL is a sulfur-free, 41‐residue SMB variant in which four cysteine and two methionine residues are replaced by tyrosines and leucines, respectively, and self‐folds through non‐covalent interactions involving its aromatic rings [11, 12]. In contrast with SMB, B-YL does not require an oxidation step to form disulfide bridges and is therefore easier and less expensive to manufacture synthetically. Excipient candidates were lactose [13] and trehalose [14] as both are commonly used in dry powder drug formulations to enhance stabilization, bulking and/or therapeutic enhancement. The non-reducing sugar trehalose was tested next to the more commonly used reducing sugar lactose, the latter of which is inclined to react with peptides. Trehalose has high water retention capabilities and preserves lipid bilayers during dehydration and rehydration by replacing water to form hydrogen bonds between its own OH groups and lipid head groups [14].

Preclinical studies in spontaneously breathing premature lambs supported with non‐invasive respiratory support (nCPAP) were based on assessments of inhaled lung dose in an in vitro lung model supported with CPAP and on surfactant efficacy and dose–response studies in ventilated, lavaged, surfactant‐deficient rabbits.

## Materials and methods

### Preparation of DP synthetic lung surfactant formulations for aerosol delivery

Four phospholipid-based DP synthetic lung surfactant preparations with a SP-B peptide mimic (SMB or B-YL) and an excipient (Lactose or Trehalose) were formulated for aerosol delivery via spray drying using alcohol-water solvent and N2 gas [8] (Table 1). The surfactant powders were formulated by adding the following components to the organic solvent used for spray drying: 49 wt.% DPPC and 21 wt.% POPG-Na (Avanti Polar Lipids, Alabaster, AL); 25 wt.% Lactose or Trehalose (Sigma-Aldrich Co, Saint Louis, MO 63103); 3 wt.% of SMB or B-YL peptide; and 2 wt.% NaCl (Sigma-Aldrich); with all components amounting to 100 wt.%. SMB and B-YL peptides were synthesized, purified using reverse phase HPLC, freeze dried, quantified, and had their mass confirmed (CSBio, Menlo Park, CA 94025) [7]. Surfactant components were mixed and spray dried to give a dry powder of respirable particle size and filled into size 00 capsules (~ 30 mg/capsule with a volume of 0.91 mL) (Acorda Therapeutics, Waltham, MA 02451, USA). Curosurf^®^ (Chiesi Farmaceutici S.p.A., Parma, Italy), a liquid porcine surfactant with native SP-B and SP-C, was used as positive control and DP surfactant without SP-B peptide mimic (DPPC:POPG:SD-30:NaCl 49:21:28:2 wt.%, [8]) as historic negative control (Lipids), where appropriate.

**Table 1:** Composition of the four experimental DP synthetic lung surfactants and controls

Abbreviated name	Components	Wt.%
*Experimental DP synthetic lung surfactant*		
SMB:Lactose Surfactant	DPPC:POPG:SMB:Lactose:NaCl	49:21:3:25:2
B-YL:Lactose Surfactant	DPPC:POPG:B-YL:Lactose:NaCl	49:21:3:25:2
B-YL:Trehalose Surfactant	DPPC:POPG:B-YL:Trehalose:NaCl	49:21:3:25:2
SMB:Trehalose Surfactant	DPPC:POPG:SMB:Trehalose:NaCl	49:21:3:25:2
*Surfactant controls*		
Positive control	Curosurf^®^	80 mg/mL
Negative controls (Lipids)	DPPC:POPG:SD-30:NaCl	49:21:28:2

### Surfactant efficacy and dose–response studies in surfactant-deficient rabbits

All animal study protocols were reviewed and approved by the Institutional Animal Care and Use Committee of the Lundquist Institute for Biomedical Innovation at Harbor-UCLA Medical Center (# 020645). Procedures and anesthesia followed American Veterinary Medical Association (AMVA) guidelines.

Juvenile male New Zealand white rabbits with a body weight of 1.0–1.2 kg were purchased from IFPS Inc. (Norco, CA 92860). Rabbits received standard veterinary care and received anesthesia with 50 mg/kg of ketamine and 5 mg/kg of acepromazine intramuscularly prior to inserting a venous line into a marginal ear vein. The venous line was used for anesthesia (maintenance with 30 mg/kg/h of propofol and 1 mg/kg of midazolam as needed) and fluid therapy (Ringer’s lactate solution). After placement of an (oro)tracheal tube (ID 3.0 mm) and a carotid arterial line via a small incision in the neck, volume-controlled ventilation (Harvard Apparatus, Holliston, MA 01746) was started with a tidal volume of 7.5 mL/kg, a peak inspiratory pressure (PIP) of 12 cm H_2_O, a positive end-expiratory pressure (PEEP) of 2 cm H_2_O, an inspiratory/expiratory ratio of 1:1, 100% oxygen, and a respiratory rate sufficient to maintain the arterial partial pressure of carbon dioxide (PaCO_2_) below 50 mmHg. Heart rate, blood pressure, and rectal temperature were monitored continuously and body temperature was maintained with a heating pad. Airway flow and pressures and (inspiratory and expiratory) tidal volume were monitored with a pneumotachograph connected to the endotracheal tube and recording system (Hans Rudolph Inc., Shawnee, KS 66227). Dynamic compliance (tidal volume/kg body weight divided by [PIP-PEEP]) was calculated and stored in conjunction with oxygenation data. Oxygenation was measured with an iStat analyzer and G3+ cartridges (Abbott POC, Princeton, NJ 08540). Oxygenation and dynamic compliance data were obtained pre-lavage (baseline), 15 and 30 min post-lavage, and 15, 30, 45, 60, 90 and 120 min after surfactant administration.

After a PaO_2_ was > 400 mmHg was established at a PIP < 15 cm H_2_O, surfactant-deficiency was achieved with repeated lung lavages of 30 mL/kg of pre-warmed normal saline (median 6 lavages) until PaO_2_ values from two arterial blood gas samples, acquired 15 and 30 min apart following final lavage to confirm stability, were < 100 mmHg (with a PIP < 25 cm H_2_O) and dynamic compliance had decreased to ≤ 50% of the baseline value. Following post-lavage measurements, rabbits were treated with surfactant. Administration of one capsule with ~ 30 mg of surfactant powder took ~ 90 s and 50 pushes in sync with spontaneous breathing with a bellows bottle or resuscitator attached to the aerosolizer.

In the surfactant efficacy study, groups of 4–5 rabbits received 4 capsules (~ 120 mg) of one of the four experimental DP surfactant powders from Table 1 or the negative control (Lipids), resuspended in dH_2_O (30 mg of DP surfactant/mL), or 200 mg/kg of the positive control (Curosurf) as a liquid bolus via a catheter with its tip located at the distal end of the endotracheal tube.

In the dose–response study, groups of 6 rabbits received 1, 2, 4 or 8 capsules of ~ 30 mg of B-YL:Lactose or B-YL:Trehalose DP surfactant by intratracheal DP aerosol delivery. Capsules were punctured with a CVT-301 device and transferred into a low flow aerosol chamber (LFAC device) connected to the swivel of the ventilator system and driven by a 30 mL bellows bottle. Completion of emission from the capsules was confirmed by weighing the capsules and the delivery system (LFAC + bellows + tubing) before and after aerosol delivery. After aerosol delivery, components were dried in an incubator at 37 °C for 1 h prior to the weight measurements. DP surfactant redosing (same surfactant preparation and same dosage) was allowed if the PaO_2_ did not increase beyond 200 mmHg within the first hour after treatment for those animals that originally received 1, 2 or 4 capsules of B-YL:Lactose or B-YL:Trehalose DP surfactant.

Rabbits were maintained for 2 h post-surfactant treatment with volume-controlled ventilation and 100% oxygen. After a blood sample was collected, the rabbits were euthanized with 200 mg/kg of pentobarbital intravenously. After euthanasia, a lung lavage was performed with 30 mL/kg of normal saline, the lungs were inspected, and biopsies of the right lower and right middle lobe were obtained. Blood, collected in serum tubes, was processed by centrifugation at 500*g* for 5 min at room temperature. Serum, bronchoalveolar lavage (BAL) fluid and lung samples were stored at − 70 °C until analysis for measurement of B-YL, DPPC and POPG concentrations.

### In vitro premature lamb lung model assessment of particle size and inhaled lung dose

Prior to the in vivo premature lamb studies, we used an in vitro premature lamb lung model set-up to measure aerosol particle size and inhaled surfactant dose and to determine the effects on lung pressure and tidal volume during simulated aerosol delivery of B-YL:Trehalose surfactant with noninvasive nCPAP support.

#### Particle sizing

A multi-staged Mini Cascade Aerosol Impactor (In-Tox Products, Clinton, MS 39056) was used to characterize distribution of dry powder aerosol particles sizes generated by LFAC and classify into respirable size fractions. The impactor uses seven individually staged particle trays with preconditioned 25 mm glass fiber filter substrates to collect aerosol. Filters were pre-conditioned with Molykote silicone spray to prevent particle bounce and recirculation, dried and weighed with an AX205 Delta Range Lab balance (Mettler-Toledo, Columbus, OH 43229). A fenestrated mixing inlet was placed at the impactor inlet to decouple flows generated by LFAC bellows (8 L/min) and maintain impactor flow (2 L/min) generated by a frit resistor (S/N 511197-9, Cole Parmer, Vernon Hills, IL 60061). Flows were confirmed with a TSI 5200 Flow and Pressure Analyzer (TSI Industries, Shoreview, MN 55126). A standard leak test was performed per manufacturer’s specifications before each test. A 3D printed nasal caste of a 28-week premature infant was connected to the mixing adaptor and impactor to capture large particles and assess nasal deposition of aerosol. The LFAC device was pre-loaded with a punctured DP B-YL:Trehalose capsule (30 mg) and attached to the nasal caste via sealed bi-nasal prongs. Powder surfactant was aerosolized through the prongs, nasal airway, and impactor by intermittently actuating a 60 mL plastic bellows attached to the distal end of the LFAC 50 times. Following nebulization (n = 6), aerosol drug mass deposited in the nasal caste airways and glass fiber filters was quantified gravimetrically based on pre/post differences in deposited mass at each location. Using the proportion of the total aerosolized mass on each stage, a log-normal distribution was fitted based on the proportion particles cut by their aerodynamic diameter. The mass median aerosol diameter (MMAD) and geometric standard deviation (GSD), of aerosolized surfactant was calculated using a log-probit equation. The fine particle fraction (FPF) was calculated as the proportion of all particles, consisting of respirable particles < 5.4 µm, delivered to the impactor stages and referenced to the emitted capsule dose.

#### Inhaled dose

We evaluated inhaled dose of aerosolized DP B-YL:Trehalose using a spontaneously breathing lung model simulator (ASL 5000, Ingmar Medical, Pittsburgh, PA 15206) that was configured based on the respiratory breathing parameters and lung mechanics of a premature (3 kg) lamb with a respiratory rate of 50/min, tidal volume of 20 mL, inspiratory time of 0.4 s, resistance of 80 cmH_2_O/L/s and compliance of 1 mL/cmH_2_O [8]. A simulated premature lamb nasotracheal airway model was attached to the lung model and bi-nasal prongs were inserted into the nasal airway openings. This prong configuration was developed so that investigators could administer aerosol through one naris without interrupting CPAP during dosing in subsequent lamb studies. Following aerosolization with this interface, the LFAC device could be removed from one prong and animals supported with CPAP delivered through bilateral prongs to maintain greater end-expiratory lung volumes and lower work of breathing than a single nasal prong. When administering aerosolized surfactant during in vitro studies, one prong was attached to LFAC and the other attached to bubble CPAP set at 6 cmH_2_O and flow of 6–8 L/min during aerosol delivery. A pre-conditioned glass fiber collection filter (47 mm) sprayed with Molykote was placed between the nasotracheal airway and lung model placed within a low dead space housing to collect aerosolized surfactant. Inspiratory pressure and tidal volume were measured within the lung model to evaluate effects of intermittent LFAC actuations and gas delivery on the lungs. A 50 mg dose of DP B-YL:Trehalose was aerosolized into the simulated airway and spontaneously breathing lung model for a total of six separate runs (n = 6). Aerosol generation with bellows was synchronized with lung model inhalation to provide a concentrated aerosol bolus during inhalation and prevent expiratory drug loss to the bubble CPAP system. Following surfactant delivery, the glass fiber filters were removed and the inhaled drug mass to the lung model was quantified gravimetrically with the lab balance. The inhaled mass delivered distal to the nasotracheal airway was then referenced as a proportion of the drug emitted from the capsule and nominal dose placed into the capsule.

### Surfactant efficacy study in premature lambs supported with nCPAP

Premature lambs at 137 days of gestation (term is 150 days) are surfactant-deficient but can be supported with noninvasive ventilation (NIV) and nCPAP via nasal prongs. General anesthesia for premature delivery by Cesarean section inhibits the start of spontaneous breathing in the fetus, so we used caudal spinal anesthesia to minimize fetal depression and optimize spontaneous breathing in the fetuses soon after birth. However, premature lambs born by Cesarean section undergo periods of hypoventilation or apnea, even if general anesthesia is avoided [15]. We therefore followed the protocol from Dargaville et al. [15] and handled hypoventilation and apneas with tactile stimulation, IV administration of doxapram and/or noninvasive ventilation (NIV) via the nasal prongs.

Date-mated pregnant ewes with singleton or twin pregnancies (a total of 20 lambs) with a gestational age of 137 days were purchased from Nebeker Ranch (Lancaster, CA 93536). Before the Cesarean section, a catheter was placed in a jugular vein of the ewe to collect heparinized blood to transfuse the lamb in case of hypotension or blood loss. Spinal anesthesia was induced with 5 mL of a 2% lidocaine solution and the uterus was exposed through a low oblique flank incision. The fetus was located and after exteriorization of its lower body, catheters were placed in an arterial and a venous umbilical vessel. After delivery of the head, a cuffed endotracheal tube (ID 3.5 mm) was inserted orally for removal of fetal lung fluid prior to cutting of the umbilical cord and subsequent short-term mechanical ventilation. The ewe was at that time euthanized with an intravenous overdose of pentobarbital.

The lamb was then dried, weighed, nursed in a radiant warmer with a heating pad and heating lamps, and started on volume-controlled ventilation (Harvard Apparatus, Holliston, MA 01746) with a tidal volume of 7 mL/kg, inspiratory: expiratory ratio of 35:65, a respiratory rate of 40 breaths/min, a PEEP of 8 cmH_2_O, and 100% oxygen. We fitted the nostrils of each lamb with an uncuffed tracheal tube (12 cm long, ID 2.5–4.0 mm) that passed the narrowing of the nose when inserted for 6–7 cm. The nasal prongs were fixed in position with superglue and attached to a Y-swivel for connection to the ventilator or CPAP system.

As soon as the lamb demonstrated spontaneous breathing activity, it was extubated and switched to NIV at the same setting via the nasal prongs. An open 10 Fr rubber gastric catheter was inserted orally to prevent nCPAP-induced abdominal bloating and the snout was wrapped in elastic bandage to prevent opening of the mouth. The ventilation rate was reduced gradually to transition to spontaneous breathing with nCPAP support, while maintaining a pH > 7.20 and PaCO_2_ < 65 mmHg. The nCPAP set-up provided heated and humidified bubble CPAP with a PEEP of 8 cm H_2_O and 100% oxygen, using a humidifier and a bubble CPAP generator (Fisher & Pykel Healthcare Inc., Irvine, CA 92618) or a Seattle bubble CPAP generator (Draeger Inc., Telford, PA 18969). About 15 min after successful transition to nCPAP, the premature lamb received aerosolized DP B-YL:Trehalose surfactant via one of the nasal prongs using the LFAC device connected to an infant resuscitator (Ambu Inc., Columbia, MD 21045, USA) fitted with a pressure-limiting valve set at 40 cm H_2_O and supplied with 2 L/min of 100% oxygen. nCPAP was continued via the other nasal prong during surfactant administration. The B-YL:Trehalose surfactant dose was ~ 120 mg (4 capsules of ~ 30 mg each)/kg body weight. If the lamb had a PaO_2_ < 200 mmHg within 2 h after the first surfactant dose, an equivalent second dose was given. Positive controls received single dose of 200 mg/kg (2.5 mL/kg) of Curosurf intratracheally prior to extubation and nCPAP as this is a liquid porcine surfactant preparation.

If necessary, spontaneous breathing was encouraged by tactile stimulation, and, in case of persistent superficial or slow breathing, by administration of 5 mg/kg of doxapram intravenously. If this intervention failed or if hypoventilation led to a drop in oxygen saturation and/or an increasing PaCO_2_, lambs were placed onto NIV with the ventilator via prongs until recovery of adequate spontaneous breathing and oxygen saturation. Pulse oximetry, heart rate, rectal temperature, and arterial blood pressure were recorded throughout the experiment. Anesthesia consisted of 2 mg/kg of propofol intravenously directly after birth, followed by 20 mg/h intravenously. Lactated Ringer’s solution was given intravenously at 10 mL/kg/h and NaCl 0.9% over the arterial line at 5 mL/kg/h. During mechanical ventilation immediately after birth, tidal volume was monitored with a pneumotachograph (Hans Rudolph Inc, Shawnee, KS 66227).

Arterial pH and blood gas measurements were obtained before and every 15–30 min after surfactant treatment. Three hours after completion of the first surfactant treatment, a blood sample was collected, and lambs were euthanized with 100 mg/kg of pentobarbital intravenously. After surgical re-insertion of an endotracheal tube (ID 3.5 mm), a standard pressure–volume curve was performed, and a lung lavage was performed with 30 mL/kg of normal saline, the lungs were inspected, and biopsies of the right lower and right middle lobe were obtained. Arterial pH, PaO_2_, and PaCO_2_ and postmortem lung compliance were used as endpoints of the experiment. Blood and lung samples were stored at − 70 °C and later used for measurements of B-YL, DPPC and POPG content.

Lung dose (mg/kg) was determined from the body weight (BW, kg), total weight of the surfactant content of the capsules (TWC, mg), the change in weight of the capsules after surfactant administration (DWC, mg), and the change in weight of the LFAC device and tubing after its use for surfactant administration (DWLFAC, mg) as follows: (TWC–DWC–DWLFAC)/BW (mg/kg). After surfactant administration, the nasal prongs were returned to their original position and function, so loss of surfactant in the nasal prongs was not accounted for.

### Measurement of DPPC, POPG, and B-YL in BAL fluid, serum and lung tissue samples

#### Sample collection and processing

Blood (1 mL) from rabbits or lambs was collected in serum tubes, centrifuged, and resultant serum was stored frozen prior to extraction and analysis. Lung (~ 1 g) from both species was also collected and stored frozen until processed for analysis. Lung tissue samples designated for analysis of DPPC or POPG were added to 450 µL of 4% bovine serum albumin (BSA) in a 1/9 ratio (w/v, tissue/BSA), homogenized using a Precellys^®^ Evolution tissue homogenizer (Bertin Corp, Rockville, MD) and centrifuged at 13,000 rpm at 2 to 8 °C for 10 min followed by analysis of the supernatant. Lung tissue designated for analysis of B-YL was thawed on wet ice and under attenuated light, rabbit or sheep lung tissue was mixed with methanol/1% formic acid/1% ascorbic acid in a 1/4 ratio (w/v, tissue/methanol with 1% formic acid and 1% ascorbic acid), homogenized using a Precellys^®^ Evolution tissue homogenizer and centrifuged at 2 to 8 °C followed by analysis of supernatant.

#### Sample extraction for analysis of DPPC, POPG, or B-YL

##### DPPC (lung supernatant, bronchoalveolar lavage, or serum)

In a low-binding extraction plate, each 25 μL aliquot of standard, Quality Control (QC) sample, or study sample was mixed with 25 μL of 4% BSA in water and 500 μL of working internal standard (0.2 μg/mL in methanol). The samples were vortexed, incubated while shaking, and centrifuged. A 20 µL aliquot of the resulting supernatant was transferred to a clean low binding autosampler plate and diluted with 1000 µL of water/methanol (40/60, v/v) with 10 mM ammonium formate]/[methanol with 10 mM ammonium formate] (80/20, v/v). The samples were vortexed and centrifuged. An aliquot was injected onto an LC–MS/MS system for analysis.

##### POPG (lung supernatant, bronchoalveolar lavage, or serum)

In a low-binding extraction plate each 25 µL or 50 µL aliquot of standard, QC sample, or study sample (25 µL of serum or BAL and 50 μL lung supernatant) was mixed with 25 µL of saturated sodium chloride solution (37%, w/v) (for serum/BAL samples only) and 500 μL of working internal standard (0.2 µg/mL in methanol). The samples were vortexed, incubated while shaking, and centrifuged. A 100 μL aliquot of the BAL or serum supernatant was transferred to a clean 96-well low-binding autosampler plate and diluted with 400 μL of [methanol with 10 mM ammonium formate]/[water/methanol (40/60, v/v) with 10 mM ammonium formate] (80/20, v/v), while a 150 µL aliquot of undiluted lung tissue supernatant was transferred to a clean 2 mL low-binding autosampler plate. An aliquot of each sample was injected onto an LC–MS/MS system for analysis.

##### B-YL (lung supernatant, bronchoalveolar lavage, or serum)

On wet ice and under attenuated light, each 50 μL aliquot of standard, QC sample, or study sample (serum or lung tissue supernatant) was mixed with 20 μL water/sodium chloride/ascorbic acid (100/31/0.2 v/w/w). The samples were vortexed and centrifuged. A 100 µL aliquot of working internal standard solution (200 ng/mL in methanol with 1% formic acid and 1% ascorbic acid) was added. The samples were vortexed and centrifuged. A 100 µL aliquot of the resulting supernatant was transferred to clean 2 mL low-binding Truetaper autosampler plate, and centrifuged. An aliquot was injected onto an LC–MS/MS system for analysis.

#### Liquid chromatography (LC)/mass spectrometry (MS) analytical conditions

##### DPPC and POPG

The liquid chromatography system used for analysis of DPPC or POPG was an XSelect-CSH Phenyl Hexyl column, 2.1 × 50 mm (3.5 μm particle size) (Waters Corporation, Milford, MA 01757) with a gradient flow consisting of water/methanol (40/60, v/v) with 10 mM ammonium formate and methanol with 10 mM ammonium formate at a flow rate of 0.500 mL/min. The analyte and internal standard (DPPC-D62) were detected using a SCIEX API 5000 triple quadrupole LC–MS/MS system (Sciex, Framingham, MA 01701) equipped with an ESI (TurboIonSpray^®^) ionization source operated in the positive ion mode. The low limit of quantitation (LLOQ) and upper limit of quantitation (ULOQ) for DPPC and POPG was 0.1 to 100 µg/mL. QC levels for DPPC or POPG included during analysis were as follows: Low QC (3 × LLOQ, 0.3 µg/mL), mid QC (approximate geometric mean to the calibration curve; 4 µg/mL), and high QC (approximately 75 to 90% of ULOQ, 80 µg/mL). A 9-point external calibration curve (0.1 to 100 µg/mL) was generated for quantitation of each analyte in study samples. Due to detectable endogenous levels of phospholipids, 4% BSA was used as a surrogate calibrant matrix for quantitation of DPPC or POPG in serum, BAL, and lung tissue supernatant.

##### B-YL

The liquid chromatography system used was a Waters XBridge Protein BEH-C4 column, 2.1 × 50 mm (3.5 μm particle size) with a gradient flow consisting of water with 1% formic acid and methanol with 1% formic acid at a flow rate of 0.500 mL/min. The analyte and internal standard (bromophenyl/quinoxaline) were detected using a SCIEX API 5000 triple quadrupole LC–MS/MS system equipped with an ESI (TurboIonSpray^®^) ionization source operated in the positive ion mode. The LLOQ for B-YL was 50 ng/mL and the ULOQ was 10,000 ng/mL. QC levels for B-YL included during analysis were as follows: Low QC (3 × LLOQ; 150 ng/mL), mid QC (approximate geometric mean to the calibration curve; 1000 ng/mL), and high QC (approximately 75 to 90% of ULOQ; 7500 ng/mL). A 9-point external calibration curve (50 ng/mL to 10,000 ng/mL) was generated for quantitation of B-YL in study samples was used as a surrogate for quantitation of B-YL in lung tissue and serum was used as a calibrant matrix for quantitation of B-YL in serum and BAL. Because B-YL is not present endogenously in study samples, QC and samples for calibration curve were prepared in serum for quantitation of B-YL in serum or BAL, and in methanol with 1% formic acid and 1% ascorbic acid for quantitation in lung tissue supernatant.

### Statistical analysis

Data are presented as mean ± SEM and/or range. Discrete data points were compared with Student’s t-test or Mann–Whitney rank sum test, whereas functional data were compared with one-way repeated measures analysis of variance with Holm–Sidak’s post-hoc test in Sigmaplot 12.3 (Systat Software Inc., Palo Alto, CA 94303). If the p value was < 0.05, a difference was determined to be statistically significant.

## Results

### Surfactant efficacy in surfactant-deficient rabbits

**Fig. 1:**
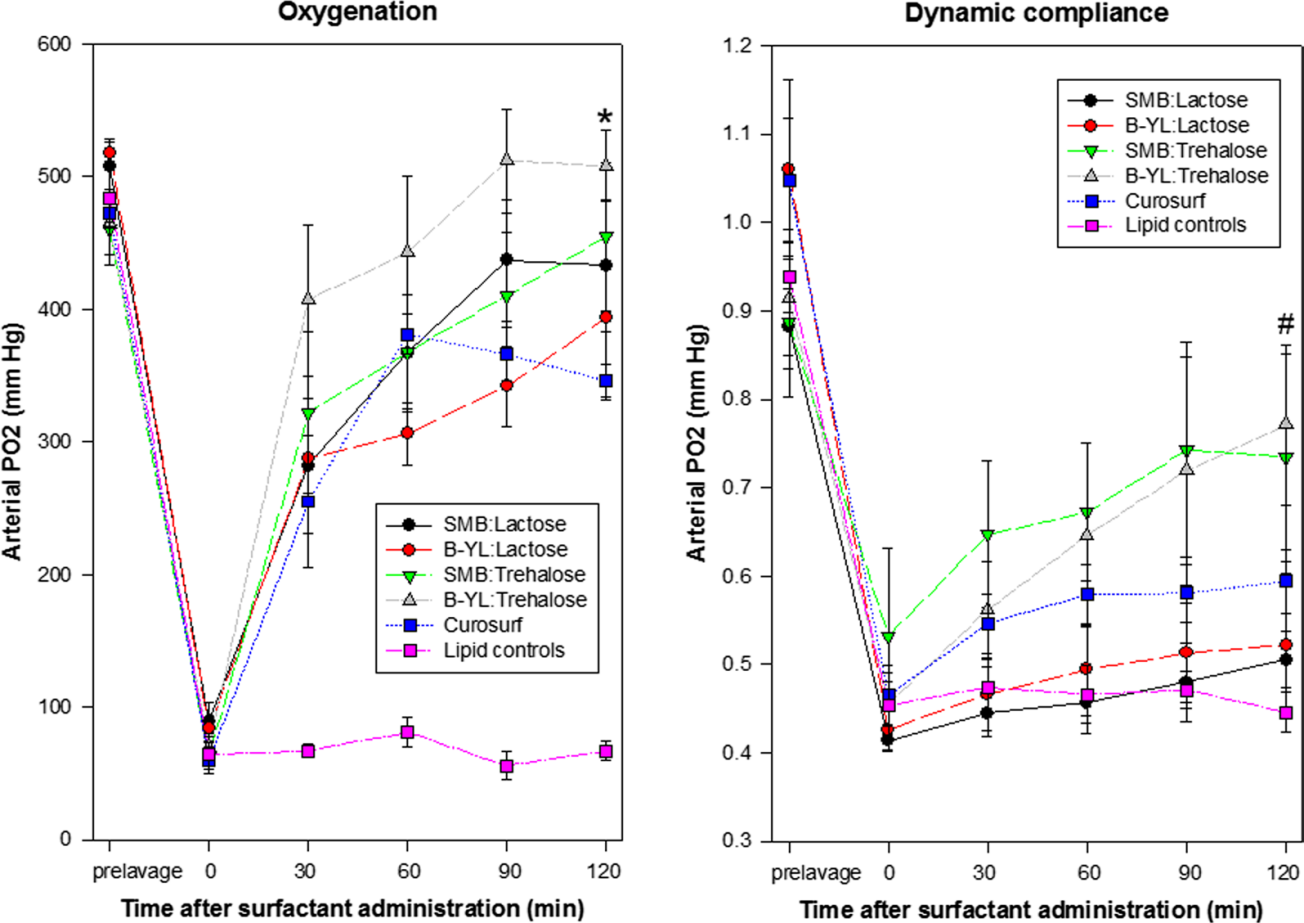
Arterial PO_2_ and dynamic compliance (mean ± SEM) in the six groups of 4–5 surfactant-deficient rabbits treated intratracheally with one of the four experimental DP synthetic lung surfactants resuspended in distilled water (30 mg/mL), the positive control Curosurf and the negative control (Lipids), described in Table 1. *p = 0.002 for B-YL:Trehalose vs Curosurf; ^#^p = 0.032 for B-YL:Trehalose vs SMB:Lactose

Twenty-six rabbits, divided over six groups of 4–5 animals, received one of the four experimental DP surfactants (Table 1), Curosurf or Lipid control. Body weights of the complete cohort were 1077 ± 15 (951–1250) g and the number of lavages needed to reach surfactant deficiency was 7.7 ± 0.7 (3–18). Baseline PaO_2_ values of the cohort were 485 ± 9 mmHg and dropped to 72 ± 4 mmHg post-lavage, whereas dynamic compliance was 0.96 ± 0.03 before and 0.46 ± 0.02 mL/kg/cmH_2_O after lavage. The surfactant dose for rabbits receiving experimental DP surfactant and Lipid control was 112 ± 2 (96–125) mg/kg, whereas the positive controls received 232 ± 8 mg/kg of Curosurf. Figure 1 demonstrates the effects of surfactant treatment on PaO_2_ and dynamic compliance in the six groups of rabbits. Intratracheal instillation of the four dissolved experimental DP synthetic lung surfactants with a SP-B peptide mimic and the clinical surfactant Curosurf induced a quick improvement in oxygenation and lung compliance and resulted in final PaO_2_ values of 425 ± 23 mmHg and a dynamic compliance of 0.64 ± 0.04 mL/kg/cmH_2_O in these merged five groups. Intratracheal treatment with the negative lipid control did not significantly improve oxygenation or dynamic compliance.

B-YL:Trehalose surfactant preparations tended to a greater response in oxygenation and dynamic lung compliance than the surfactant preparations with lactose as excipient (Fig. 1). Significant differences in oxygenation were only found between B-YL:Trehalose and Curosurf (p = 0.002) and in dynamic compliance between B-YL:Trehalose and SMB:Lactose (p = 0.032) at 2 h post-surfactant. When the data of SMB:Lactose and B-YL:Lactose were combined and compared to the combination of SMB:Trehalose and B-YL:Trehalose, post-lavage oxygenation and dynamic compliance data were comparable, post-surfactant oxygenation increased similarly, but dynamic compliance increased significantly faster in the Trehalose than in the Lactose surfactant groups (p < 0.04 at 30, 60, 90, and 120 min post-surfactant).

### Surfactant dose–response in surfactant-deficient rabbits

Groups of 6 surfactant-deficient rabbits were treated with 30, 60, 120 or 240 mg/kg of DP B-YL:Trehalose or B-YL:Lactose surfactant by intratracheal aerosol delivery using the LFAC device. Birth weight of this cohort was 1085 ± 11 g and initial PaO_2_ and dynamic compliance values were 500 ± 5 mmHg and 0.87 ± 0.02 mL/kg/cm H_2_O. After repeated lung lavages (5.3 ± 0.4, range 2–12), PaO_2_ and dynamic compliance values were 60 ± 3 mmHg and 0.38 ± 0.01 mL/kg/cm H_2_O.

**Fig. 2:**
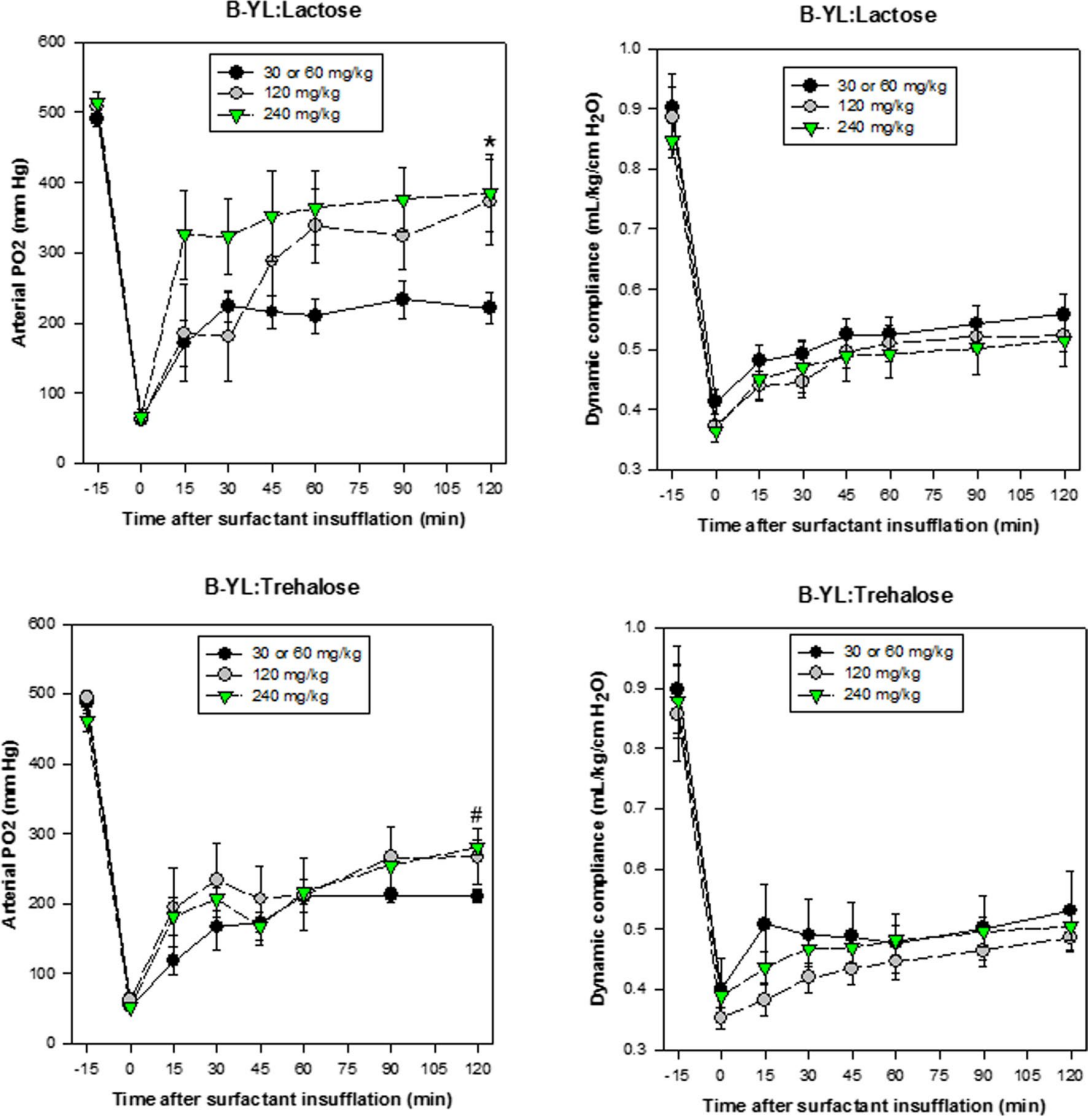
Oxygenation (PaO_2,_ mmHg) and dynamic compliance (mL/kg/cm H_2_O) (mean ± SEM) of surfactant-deficient rabbits treated with intratracheal aerosol delivery of dry powder B-YL:Lactose (top) or B-YL:Trehalose (bottom) surfactant. Animals are sorted according to the total dose of surfactant administered over 1 or 2 doses. *p = 0.045, respectively 0.015, for B-YL:Lactose 30 or 60 vs 120 and 240 mg/kg; ^#^p = 0.03, respectively 0.015, for B-YL:Trehalose 30 or 60 vs 120 and 240 mg/kg at 120 min post-surfactant

Figure 2 shows the changes in oxygenation and dynamic compliance after intratracheal insufflation of 30, 60, 120 or 240 mg/kg of DP B-YL:Lactose and B-YL:Trehalose surfactant. All dosing regimens led to an improvement in PaO_2_, but 120 and 240 mg/kg resulted in higher PaO_2_ values than 30 and 60 mg/kg. The data from the 30 and 60 mg/kg groups were combined because frequent necessity of retreatment led to smaller numbers of rabbits in these lower dosage groups. In the B-YL:Lactose group, 3 rabbits received 30 mg/kg group, 6 received 60 mg/kg, 7 received 120 mg/kg, and 8 received 240 mg/kg of surfactant. Differences in end-terminal PaO_2_ values at 120 min post-surfactant between the combined 30 and 60 mg/kg group and the 120 and 240 mg/kg groups and the combined 120 and 240 mg/kg group were statistically significant (p = 0.045, 0.015 and 0.02). In the B-YL:Trehalose group, 5 rabbits received 30 mg/kg, 1 got 60 mg/kg, 8 got 120 mg/kg, and 10 got 240 mg/kg of B-YL:Trehalose. Here, the differences between the combined 30 and 60 mg/kg group and the 120 and 240 mg/kg groups and the combined 120 and 240 mg/kg group were also statistically significant (p = 0.03, 0.015 and 0.005) at 120 min post-surfactant. These findings suggest that 120 or 240 mg/kg of DP synthetic lung surfactant was more effective in improving oxygenation than a dose of 30 or 60 mg/kg.

Dynamic compliance (mL/kg/cmH_2_O) increased in all treatment subgroups with ~ 25% recovery of the post-lavage values after B-YL:Trehalose or B-YL:Lactose surfactant treatment. The differences between the 30, 60, 120 and 240 mg/kg dosing regimens did not reach statistical significance as both surfactant formulations and all dosages responded similarly with a slow and steady response that was not clearly dose-dependent.

We measured the amount of surfactant delivered with the LFAC device by weighing the application system before and after treatment. Combining the data of all 48 rabbits, we found that on average 71.5 ± 0.7% of the projected dose was delivered to the animals. About half of the loss was due to incomplete emptying of the capsules and the other half of the loss was secondary to retention in the LFAC device and swivel connected to the endotracheal tube.

**Fig. 3:**
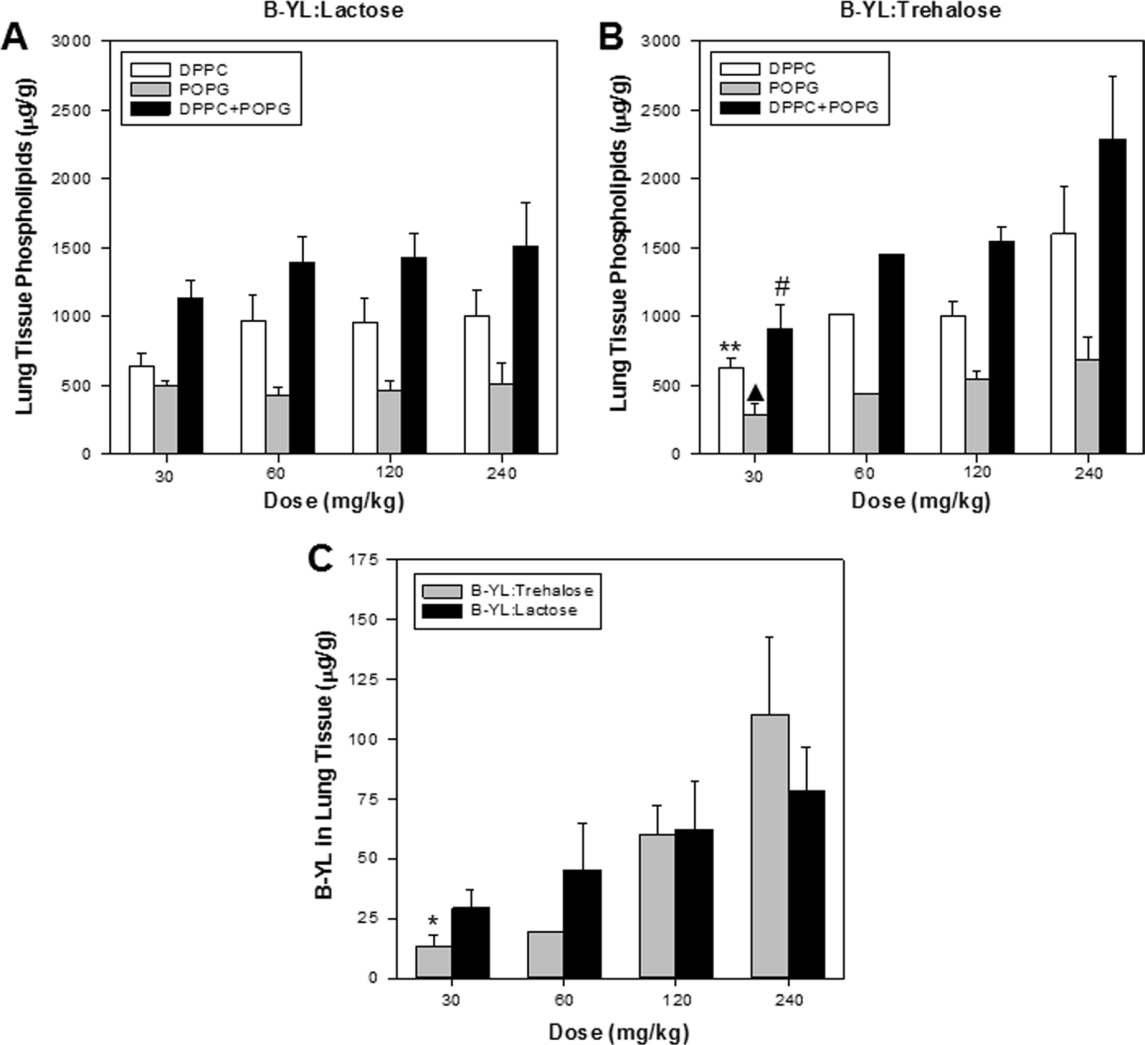
Phospholipid (DPPC and POPG) and B-YL concentrations (mean ± SEM) in lung tissue (µg/g) from surfactant-deficient rabbits measured 2 h after intratracheal insufflation of dry powder. **A** Lung DPPC and POPG concentrations after aerosol delivery of DP B-YL:Lactose surfactant. **B** Lung DPPC and POPG concentrations after DP B-YL:Trehalose surfactant treatment. **C** B-YL peptide concentration in lung tissue after B-YL:Lactose and B-YL:Trehalose surfactant treatment. *p = 0.0285 for B-YL 30 vs 120 mg/kg; **p = 0.0348 for DPPC 30 vs 120 mg/kg; ^▲^p = 0.0385 for POPG 30 vs 120 mg/kg; ^#^p = 0.0080 for DPPC + POPG 30 vs 120 mg/kg. Lower limit of quantitation (LLOQ) was 0.1 µg/mL for DPPC and POPG and 50 ng/mL for B-YL

Surfactant delivery efficacy was also determined by measuring phospholipid (DPPC and POPG) and B-YL peptide concentration in rabbit lung tissue and in serum (data not shown). Figure 3 shows a dose-related increase in concentrations of B-YL, DPPC (p = 0.048) and DPPC + POPG (p = 0.036) in lung tissue with increasing dosages of B-YL:Trehalose or B-YL lactose confirming a lung exposure/efficacy response. The total lung concentrations of DPPC + POPG and B-YL reached maximal mean concentrations of 2289 µg/g and 110 µg/g, respectively. Although concentrations of these analytes were not measured in untreated surfactant-deficient rabbits, endogenous concentrations of DPPC or POPG in lungs from untreated normal rabbits were approximately 100 µg/g, which is still 20-fold lower than concentrations measured in treated surfactant-deficient rabbits confirming significant elevations following treatment. Endogenous levels of the peptide B-YL was not detected in untreated normal lung tissues. Following surfactant treatment, serum concentrations of B-YL, DPPC and POPG did not increase with treatment and were within limits of quantitation of the respective assays confirming lack of systemic exposure of any analyte in blood following intratracheal administration of the surfactant.

Although increased phospholipids were detected above endogenous concentrations, high background phospholipids in lung tissue limited interpretation of composition of DPPC:POPG in biological samples compared to administered levels. Because rabbits were rendered surfactant deficient prior to administration of exogenous surfactant, they provided a cleaner model for analysis of phospholipids in terminal lavage samples for an estimation of exposure relative to administered surfactant. Table 2 summarizes concentrations of DPPC, POPG, and B-YL measured in terminal lavage samples from rabbits. DPPC, POPC and B-YL increased with dose, but more importantly the DPPC:POPG ratios of 2.3:1 to 5.5:1 approached levels administered (2.3:1 or 7:3), especially at the higher doses of 120 and 240 mg/kg confirming retention of composition of surfactant dosed.

**Table 2:** Mean concentrations of DPPC, POPG, and B-YL in Bronchoalveolar Lavage samples from rabbits treated with B-YL:Trehalose or B-YL:Lactose

Dose (mg/kg)	DPPC (µg/mL)	POPG (µg/mL)	B-YL (µg/mL)	DPPC:POPG
30	173 ± 66	31.3 ± 9.1	6.85 ± 1.7	5.5:1 (16:3)
60	332 ± 31	71.0 ± 48	7.70 ± 4.7	4.7:1 (15:3)
120	590 ± 114	238 ± 79	14.1 ± 2.3	2.5:1 (7:3)
240	665 ± 121	278 ± 98	21.9 ± 4.5	2.4:1 (7:3)

Data are represented as mean ± SEM, n = 6–15 per group

### In vitro premature lamb lung model assessment of particle sizing and inhaled lung dose

Using the spontaneously breathing premature lamb lung model simulator set-up, surfactant particles generated by LFAC delivery had a MMAD of 3.6 µm and GSD of 1.8. The nasal deposition of aerosol was 18 ± 3% of the emitted capsule dose. The FPF of aerosol (< 5.4 µm) was 94 ± 6% when referenced to the total mass of aerosol delivered to the impactor. The inspiratory pressure and volumes delivered to the lung model increased 1.1 ± 0.9 cmH_2_O and 1.7 ± 0.4 mL, respectively above baseline breathing condition when bellows actuation of LFAC was used for aerosol delivery. The inhaled mass of aerosolized surfactant delivered distal to the nasotracheal airway was 66.8 ± 7% and 53.3 ± 13% of the emitted and nominal dose, respectively. Thus, a significant proportion of small aerosol particles (65%) emitted by LFAC could be safely delivered into the distal airways during simulated aerosol administration with CPAP.

### Aerosol delivery of DP B-YL:Trehalose surfactant in premature lambs

Twenty premature lambs transitioned from mechanical ventilation to nCPAP with 100% oxygen, 11 were males and 9 females, 12 were singletons and 8 twins, and birth weight was 3.1 ± 0.1 kg. Lambs were allocated to 3 treatment groups: (1) eight lambs received one dose of B-YL:Trehalose surfactant; (2) nine lambs received two doses of B-YL:Trehalose surfactant, and (3) three lambs were treated with Curosurf as positive controls. The choice between one or two doses of B-YL:Trehalose surfactant was based on the course of oxygenation, i.e. a PaO_2_ value ≤ 200 mmHg during the first 2 h after the first dose of surfactant was an indication for a second identical dose of surfactant. All lambs that received treatment completed the study protocol. Two lambs who could not be resuscitated after birth were used for bioanalytical analysis.

**Fig. 4:**
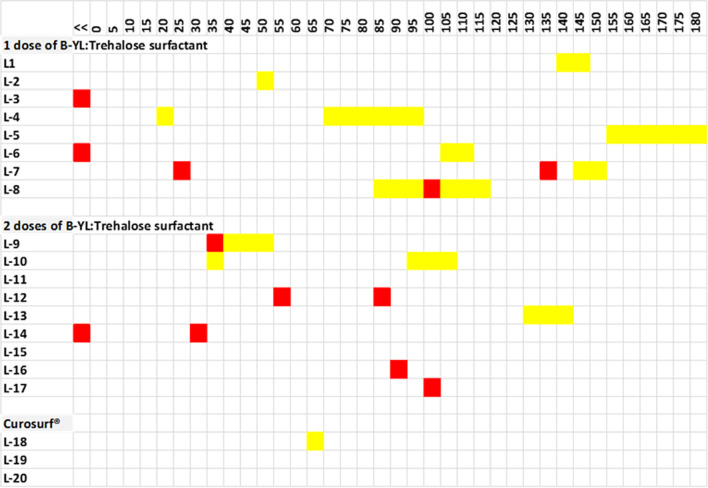
Rescue sequence of medical treatment (doxapram in red) and nasal mechanical ventilation (NIV in yellow) during the 180 min period starting after completion of (first) surfactant treatment (t = 0), divided into 5 min intervals

Nine out of the 20 lambs were able to breathe spontaneously with bubble nCPAP support during the whole 180 min post-surfactant period. In 4 out of these 9 lambs, medical support (doxapram intravenously) was needed to avoid a return to NIV. Among the 11 lambs that required one or more periods of NIV support, 4 also received medical support to stimulate breathing. Figure 4 shows that the 1-dose B-YL:Trehalose group needed more often (p = 0.024) and, on average, longer NIV than the 2-dose B-YL:Trehalose group. In the Curosurf group, one lamb needed to return to NIV support for 15 min because of increasing PaCO_2_. There was a lower pH in the Curosurf treated lambs that was not affected by PaCO_2_, but due to metabolic acidosis.

**Fig. 5:**
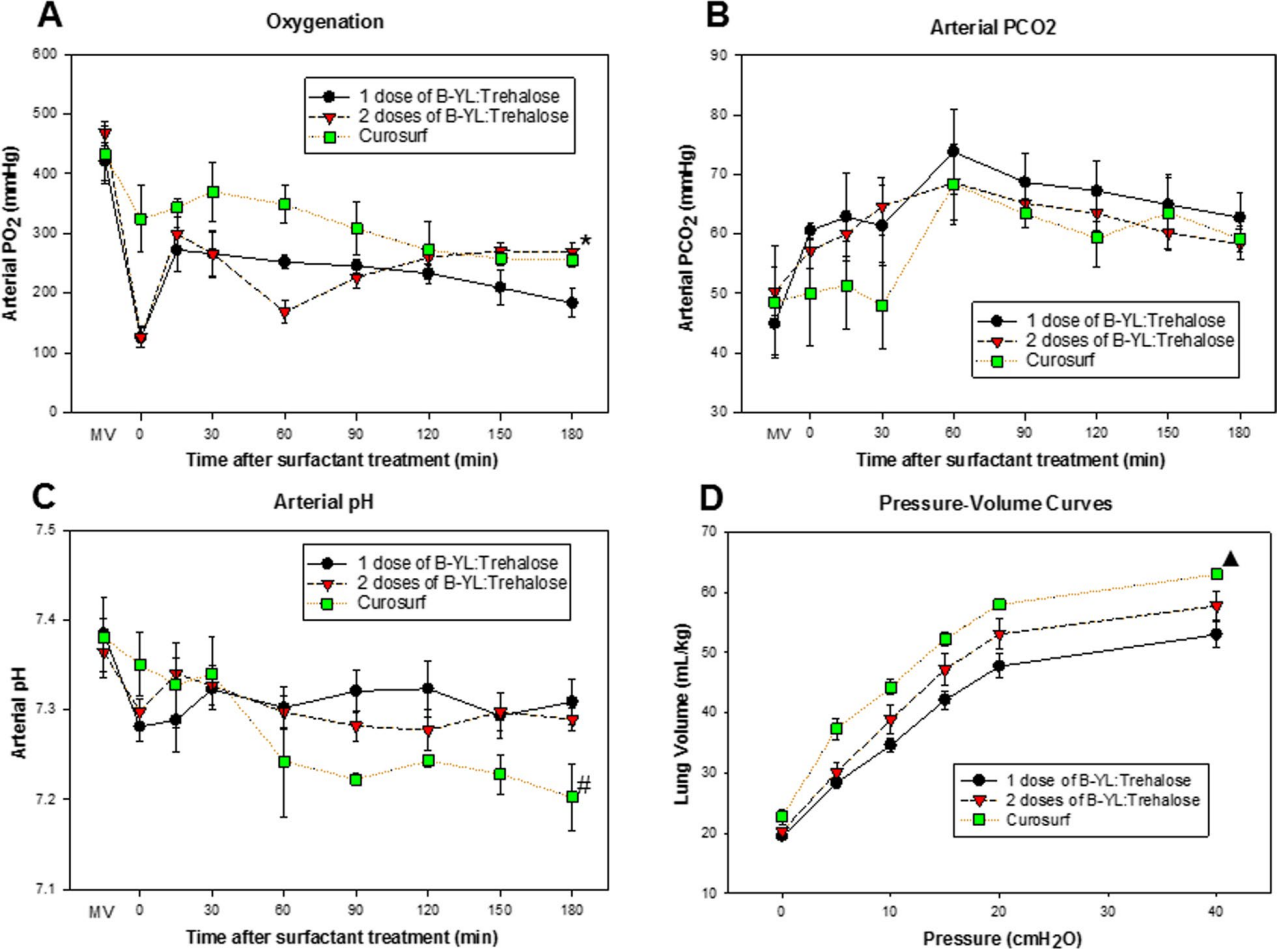
Arterial pH, partial arterial oxygen pressure (PaO_2_), and partial arterial carbon dioxide pressure (PaCO_2_), and lung volume (mean ± SEM) in the three groups of premature lambs supported with nCPAP and 100% oxygen and treated with 1 or 2 doses of aerosolized B-YL:Trehalose surfactant or the positive control Curosurf. Data reflect the period from birth until 180 min post-surfactant treatment for PaO_2_ (**A**), PaCO_2_ (**B**), and pH (**C**), whereas lung volumes were derived from postmortem pressure–volume curves (**D**). MV: mechanical ventilation. *p < 0.02 for 2-dose B-YL:Trehalose and Curosurf groups vs the 1-dose B-YL:Trehalose group; #p = 0.045 for the Curosurf vs the 1- and 2-dose B-YL:Trehalose groups; ^▲^p = 0.012 for Curosurf vs the 1-dose BYL:Trehalose group

Two doses of B-YL:Trehalose surfactant, given within two hours from each other, led to a PaO_2_ similar to that of the clinical surfactant Curosurf (268 ± 17 vs 255 ± 12 mmHg), but even one dose was able to significantly improve the PaO_2_ from starting values of 125 ± 17 to 183 ± 24 mmHg (Fig. 5A). The dip in PaO_2_ values at 60 min after the first surfactant administration in the 2-dose B-YL:Trehalose group corresponds with the lack of response of these lambs to initial surfactant treatment with a PaO_2_ ≤ 200 mmHg, thereby qualifying these animals to receive a second dose of surfactant. This approach imitates current clinical practice to administer a second dose of liquid surfactant to premature infants who do not respond sufficiently to the first dose. The second dose of surfactant increased their PaO_2_ to values similar to those of the positive control Curosurf. At 180 min post-surfactant, the differences between the 2-dose B-YL:Trehalose and Curosurf groups versus the 1-dose B-YL:Trehalose group were statistically significant (p < 0.02), suggesting a better treatment response of two than of one dose of B-YL:Trehalose surfactant. PaCO_2_ values showed relatively large upward swings due to periods of superficial or slow breathing (Fig. 5B). Treatment of these temporary increases in PaCO_2_ with respiratory stimulants and/or NIV (Fig. 4) were followed by reductions in PaCO_2_. At 180 min post-surfactant, the differences in PaCO_2_ between the three groups were not statistically significant. Arterial pH values decreased quickly after the transition to spontaneous breathing with nCPAP support but stabilized after surfactant treatment (Fig. 5C). pH values at 0 min and 180 min post-surfactant treatment were quite similar for the 1- and 2-dose B-YL:Trehalose groups, whereas those for the Curosurf group decreased after 30 min and continued to be lower than the B-YL:Trehalose groups from 60 min onwards. At 180 min post-surfactant, the lambs in the Curosurf group were significantly more acidotic than the two B-YL:Trehalose groups (p = 0.045). Pressure–volume curves demonstrated that lung volumes at 40 cmH_2_O pressure were significantly higher in the Curosurf group than in the 1-dose BYL:Trehalose group (p = 0.012) with an intermediate position for the 2-dose BYL:Trehalose group, indicating a functional advantage for the 2-dose BYL:Trehalose regimen (Fig. 5D).

Emitted surfactant dose, i.e. the actual amount of surfactant delivered via the nasal route, was derived from the weight changes of the capsules and the aerosol device after B-YL:Trehalose surfactant administration. On average, 17% of the intended dose was retained in the capsules and 12% in the LFAC device, suggesting a delivery of 71% of the intended dose to the nasal prongs. Humidification and heating of oxygen flow to the animals contributed significantly to the incomplete emptying of the capsules and loss in the LFAC device due to the hygroscopic nature of the dry powder synthetic surfactant (“agglomeration”). The intended dose was four capsules of ~ 30 (28–32) mg/kg body weight, i.e. about 120 mg/kg. Actual dosing of B-YL:Trehalose surfactant (134 mg/kg) was slightly higher than the intended dose of 120 mg/kg, but delivery losses reduced this to an average delivered dose of 97 mg/kg.

**Fig. 6:**
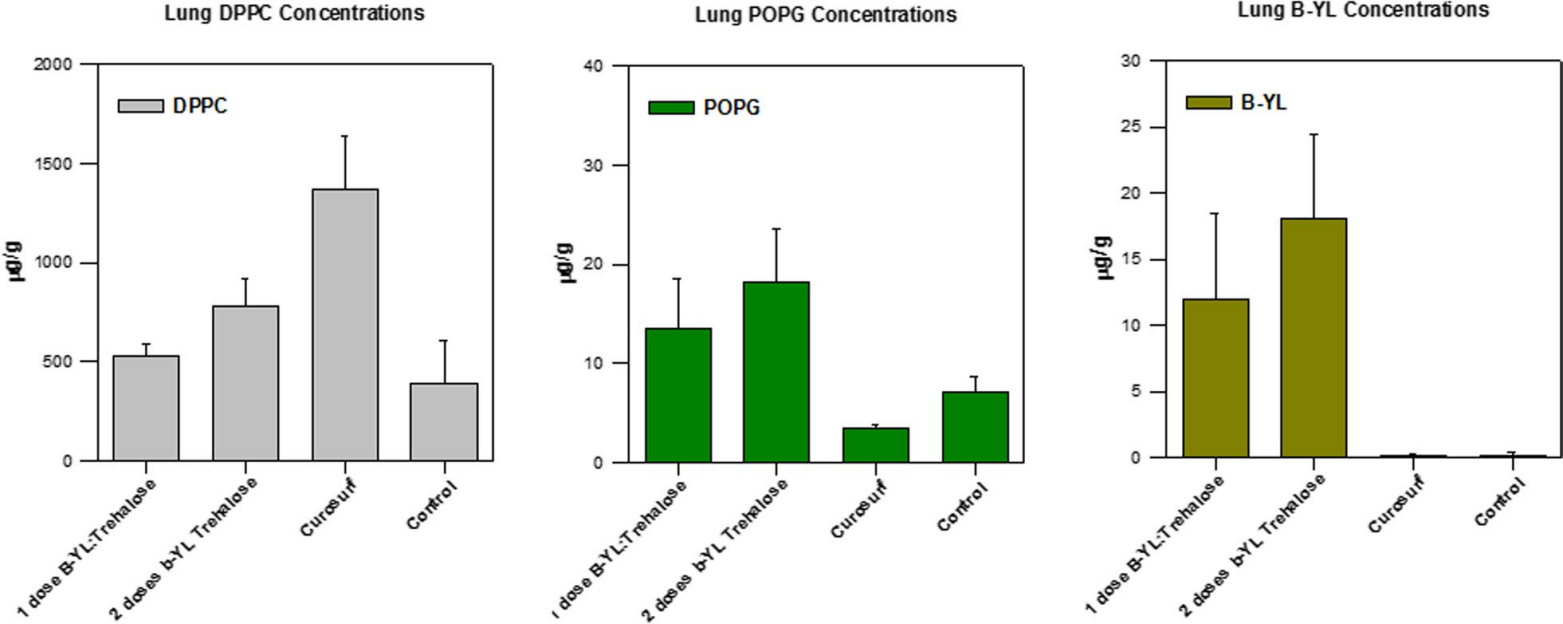
Mean ± SEM phospholipid (DPPC and POPG) and B-YL peptide concentrations in lung tissue of premature lambs treated with aerosol delivery of 1 or 2 doses of dry powder B-YL:Trehalose surfactant, intratracheal instillation of one dose of the positive control Curosurf, or untreated controls. *p ≤ 0.04 for 1- and 2-dose B-YL:Trehalose vs Curosurf; ^#^p = 0.03 Curosurf vs controls

Figure 6 presents phospholipid (DPPC and POPG) and B-YL peptide concentrations in lung tissue. Endogenous concentrations of POPG measured in lung from preterm lambs was almost two orders of magnitude lower than levels of DPPC. POPG increased about fivefold following administration of B-YL:Trehalose, while levels following administration of Curosurf did not change and were generally comparable to endogenous concentrations in untreated lambs. DPPC concentrations in lung increased with exposure to aerosolized B-YL:Trehalose surfactant, but levels were significantly lower than in preterm lambs treated with intratracheal Curosurf (p = 0.004 for 1 dose and p = 0.040 for 2 doses of B-YL:Trehalose vs Curosurf). Significant increases in concentrations of B-YL were observed only in groups receiving 1 or 2 doses of B-YL:Trehalose. B-YL and POPG were not detected in serum of treated preterm lambs, while DPPC concentrations did not increase and were within the limits of quantitation of the assay, confirming lack of systemic exposure of any analyte in blood following surfactant treatment.

## Discussion

The use of DP surfactant formulations with SMB or B-YL as SP-B peptide mimics and lactose or trehalose as excipient in this study was based on in vitro surface activity, particle sizing, and biophysical stability measurements in a large series of DP surfactant formulations with various SP-B peptide mimics and excipients that indicated greater in vitro stability and surface activity of B-YL:Trehalose surfactant. The results described here also demonstrate the efficacy of B-YL:Trehalose surfactant when administered in vivo in surfactant-deficient rabbits and premature lambs.

The efficacy study in ventilated surfactant-deficient rabbits showed that oxygenation improved much better after intratracheal instillation of dissolved SMB:Trehalose and B-YL:Trehalose surfactant powders than after administration of SMB:Lactose and B-YL:Lactose surfactant formulations. Dose–response studies with intratracheal aerosol delivery of B-YL:Lactose and B-YL:Trehalose surfactants indicated that a minimum of 120 mg/kg and preferably 240 mg/kg was needed to optimize lung function recovery in ventilated 1-kg surfactant-deficient rabbits. The dosage findings from intubated and ventilated rabbits were then used in the premature lambs who received 1 or 2 doses of 120 mg/kg body weight of B-YL:Trehalose surfactant by intranasal aerosol delivery to stabilize oxygenation and lung volume. Remarkably, the 2-dose regimen of intranasal aerosol delivery of DP B-YL-Trehalose surfactant, i.e., ~ 200 mg/kg, improved lung function almost similar to the positive control, clinical surfactant Curosurf. Another difference was seen in the frequency of rescue interventions between arms of this study, as the B-YL:Trehalose arms needed much more frequent rescue interventions than the Curosurf animals. This difference may be due to the more complex and time-consuming aerosol delivery of B-YL:Trehalose surfactant compared to the direct intratracheal delivery of Curosurf. Emitted dose in surfactant-deficient rabbits and premature lambs was about 70% due to an equivalent loss of powder in the application system and agglomeration of the powder in the capsules as a result of exposure to humidified gases.

Lung concentrations of B-YL at the minimal efficacious dose of 30 mg/kg in rabbits treated with B-YL:Trehalose or Lactose was 20.1 µg/mL, which was generally similar to mean concentrations of 18.1 µg/mL measured in premature lambs at the efficacious dose of 240 mg/kg (2 doses), providing a threshold concentration of B-YL that may mediate efficacy in preterm infants assuming a reasonable nonclinical to clinical translation. Surfactant delivery in lungs was not associated with any toxicities of concern in rabbits and lambs up to the highest dose tested of 240 mg/kg in both species. In rabbits, B-YL concentrations in lungs at the highest dose was approximately 90 µg/mL, providing a wide range of efficacious concentrations without safety issues.

At present, surfactant treatment of premature infants with respiratory distress syndrome (RDS) depends on intratracheal instillation, either by tracheal intubation in combination with mechanical ventilation or by less invasive surfactant administration (LISA) via a thin intratracheal catheter during NIV [7]. However, efficacy of aerosol delivery of surfactant depends not only on the composition of the surfactant formulation, its safety and efficacy, but also on the type of aerosolization device and non-invasive interface used, and the possibility to synchronize surfactant delivery with spontaneous breathing [16]. Three recent clinical studies have shown that non-invasive aerosol delivery of animal-derived surfactant can prevent the need for early intubation [17–20]. Synthetic lung surfactant formulations that are in clinical development include CHF5633 (Chiesi Farmaceutici, Parma, Italy) for premature infants [21, 22], recombinant SP-C for acute respiratory distress syndrome (rSP-C33Leu) [23], a peptoid-based SP-C analogue [24], and Aerosurf (Windtree Therapeutics, Warrington, PA 1897619) [24]. CHF5633, a combination a SP-B and SP-C peptide mimics mixed in phospholipids, has shown to be safe after intratracheal delivery in premature infants [20, 21]. Aerosurf consists of lyophilized KL4 surfactant (Surfaxin^®^) and is being tested for non-invasive aerosol delivery, but it has the disadvantage that it needs to be pre-heated before inhalation [25]. The newer DP synthetic lung surfactant formulations used in this study are highly surface active, possess the required aerosolization characteristics, and are biophysically stable [10]. Furthermore, when used in combination with a LFAC aerosolization device, they are effective in resolving surfactant deficiency in ventilated, lavaged rabbits and spontaneously breathing premature lambs supported with nCPAP.

## Conclusion

In summary, these studies demonstrate in vivo efficacy of DP synthetic lung surfactant with B-YL peptide as a SP-B mimic and Trehalose as excipient to alleviate surfactant deficiency in relevant preclinical models when delivered as a liquid bolus or as a dry-powder aerosol.

## Data Availability

Raw data on B-YL surfactant are available on OSF: osf.io/em53s.

## Abbreviations

AMVA: American Veterinary Medical Association
BAL: Bronchoalveolar lavage
BSA: Bovine serum albumin
BW: Body weight
CPAP: Continuous positive airway pressure
DP: Dry powder
DPPC: Dipalmitoylphosphatidylcholine
DWC: Change in weight of the capsules
DWLFAC: Change in weight of the LFAC device and tubing
FPF: Fine particle fraction
GSD: Geometric standard deviation
HPLC: High-performance liquid chromatography
ID: Internal diameter
LC–MS/MS: Liquid chromatography–mass spectrometry
LFAC: Low flow aerosolization chamber
LISA: Less invasive surfactant administration
LLOQ: Low limit of quantitation
MMAD: Mass median aerosol diameter
NaCl: Sodium chloride
nCPAP: Nasal CPAP
NIV: Noninvasive ventilation
PaCO_2_: Partial carbon dioxide pressure
PaO_2_: Partial oxygen pressure
PEEP: Positive end-expiratory pressure
PIP: Peak inspiratory pressure
POPG: 1-Palmitoyl 2-oleyl phosphatidylglycerol
SMB: Super Mini-B
SP-B: Surfactant protein B
SP-C: Surfactant protein C
QC: Quality Control
TWC: Total weight of capsule
ULOQ: Upper limit of quantitation
